# Protein kinase C theta is required for efficient induction of IL-10-secreting T cells

**DOI:** 10.1371/journal.pone.0171547

**Published:** 2017-02-03

**Authors:** Graham J. Britton, Ruth E. Mitchell, Bronwen R. Burton, David C. Wraith

**Affiliations:** School of Cellular and Molecular Medicine, University of Bristol, Bristol, United Kingdom; University of Lisbon, PORTUGAL

## Abstract

Secretion of interleukin-10 (IL-10) by CD4^+^ T cells is an essential immunoregulatory mechanism. The work presented here assesses the role of the signaling molecule protein kinase C theta (PKCθ) in the induction of IL-10 expression in CD4^+^ T cells. Using wildtype and PKCθ-deficient Tg4 T cell receptor transgenic mice, we implemented a well-described protocol of repeated doses of myelin basic protein (MBP)Ac1-9[4Y] antigen to induce Tr1-like IL-10^+^ T cells. We find that PKCθ is required for the efficient induction of IL-10 following antigen administration. Both serum concentrations of IL-10 and the proportion of IL-10^+^ T cells were reduced in PKCθ-deficient mice relative to wildtype mice following [4Y] treatment. We further characterized the T cells of [4Y] treated PKCθ-deficient Tg4 mice and found reduced expression of the transcription factors cMaf, Nfil3 and FoxP3 and the surface receptors PD-1 and Tim3, all of which have been associated with the differentiation or function of IL-10^+^ T cells. Finally, we demonstrated that, unlike [4Y] treated wildtype Tg4 T cells, cells from PKCθ-deficient mice were unable to suppress the priming of naïve T cells *in vitro* and *in vivo*. In summary, we present data demonstrating a role for PKCθ in the induction of suppressive, IL-10-secreting T cells induced in TCR-transgenic mice following chronic antigen administration. This should be considered when contemplating PKCθ as a suitable drug target for inducing immune suppression and graft tolerance.

## Introduction

An escalating dose of high-affinity myelin basic protein (MBP)-Ac1-9[4Y] peptide, administered subcutaneously (s.c.) to T cell receptor (TCR) transgenic Tg4 mice, induces peripheral tolerance characterized by the induction of IL-10-secreting CD4^+^ T cells [1]. These IL-10^+^ T cells differentiate along the T helper 1 (Th1) pathway, but convert to a regulatory phenotype as part of a negative-feedback loop maintaining peripheral tolerance [2]. Several factors including peptide affinity, dose and solubility and the route of administration used have been shown to play an important role in the successful induction of IL-10^+^ T cells [3].

In CD4^+^ T cells protein kinase C theta (PKCθ) is phosphorylated and activated following ligation of the TCR by peptide-MHC. Active PKCθ is required for initiation of NFκB-dependent transcription therefore naïve CD4^+^ T cells from PKCθ deficient mice fail to proliferate in response to antigen [4]. PKCθ has also been shown to be specifically required for the differentiation of autoimmunity-associated T helper 17 (Th17) cells [5] and to inhibit the function of FoxP3^+^ regulatory T cells (Treg) [6]. These observations have led some to suggest that PKCθ represents an attractive drug target for inducing immune suppression in the context of transplant and autoimmunity [7–9]. However, recent trials of the PKCθ inhibitor sotrastaurin (AEB071), in renal transplant patients, have demonstrated inferior efficacy compared to existing regimes [10,11].

As peptide affinity, and thus strength-of-signal from the TCR, are crucial in determining efficiency of induction of IL-10-secreting T cells from Th1 effector cells [12,13], we hypothesized that altering TCR-mediated signaling by disrupting PKCθ would impact the generation of IL-10^+^ T cells, and thus the maintenance of peripheral tolerance, following therapeutic peptide administration. We utilized PKCθ-deficient Tg4 mice (Tg4^KO^) to study the role of this signaling pathway on the generation of IL-10-secreting T cells and the induction of a tolerant immune environment following MBP-Ac1-9[4Y] administration.

## Materials and methods

### Ethical statement

All animal experiments were carried out under the UK Home Office Project Licence number 30/2705 held by David Wraith and the study was approved by the University of Bristol ethical review committee.

### Materials

The acetylated N-terminal peptides of myelin basic protein, MBPAc1-9 [4K] (AcASQKRPSQR) and [4Y] (AcASQYRPSQR) were synthesized by GL Biochem Shanghai. *In vitro* stimulations and assays were performed in complete RPMI (Lonza, supplemented with 5% fetal bovine serum (Biosera), 20mM HEPES, 2mM L-glutamine, 100U/ml penicillin, 100μg/ml streptomycin and 50mM 2-mercaptoethanol). A list of antibodies and details of their use in this study can be found in Table 1.

**Table 1:** Antibodies used in this study.

Epitope	Clone	Conjugation	Supplier	Working concentration
CD4	GK1.5	Alexa700	Biolegend	2 μg ml^-1^
IL-10	ES5-16E3	APC	eBioscience	1 μg ml^-1^
IFNγ	XMG1.2	PerCP-Cy5.5	eBioscience	0.5 μg ml^-1^
IL-4	11B11	PE-Cy7	eBioscience	0.5 μg ml^-1^
IL-17A	TC11-18H10.1	PE	eBioscience	1 μg ml^-1^
IL-2	JES6-5H4	eFluor450	eBioscience	2 μg ml^-1^
GM-CSF	MP1-22E9	FITC	eBioscience	2 μg ml^-1^
cMaf	SYMOF1	eFluor660	eBioscience	2 μg ml^-1^
FoxP3	FJK-16S	eFluor450	eBioscience	2 μg ml^-1^
Lag3	C9B7W	APC	eBioscience	2 μg ml^-1^
TIGIT	1G9	APC	Biolegend	2 μg ml^-1^
PD-1	29F.1A12	PE-Cy7	Biolegend	1 μg ml^-1^
Tim3	8B.2C12	PE	eBioscience	1 μg ml^-1^
CD3ε	2C11	None	eBioscience	1 μg ml^-1^
CD28	37.51	None	eBioscience	2 μg ml^-1^

APC–allophycocyanin.

FITC—fluorescein isothiocyanate.

PE–phycoerythrin.

PerCP—peridinin chlorophyll protein complex.

### Mice and peptide treatments

PKCθ-deficient Tg4 mice were generated by cross breeding Tg4 mice [14] with B6.129P2-*Prkcq*^*tm1Litt*^/J mice [4] (originally generated by D. Littman, a gift of A. Poole, Bristol) for at least 8 generations. Expression of the transgenic Vβ8 TCR and H-2^u^ was confirmed by flow cytometry and deletion of PKCθ was assessed by PCR of genomic DNA isolated from tail or ear tissue as described [4]. Tg4^WT^, Tg4^KO^ and B10.PL mice were bred and maintained under SPF conditions at the University of Bristol with constant access to water and standard lab chow. Male and female mice aged 6–14 weeks were used and were equally distributed between groups based on age and sex. Mice were injected s.c. with PBS or [4Y] peptide in a volume of 200μl of sterile PBS every 3–4 days. [4Y] doses were increased incrementally (0.08, 0.8, 8, 80, 80, 80μg) as previously described [1]. All *ex vivo* analyses were performed 2 hours after the final dose of peptide.

### Serum cytokine measurements

Peripheral blood samples were taken from the tail vein of mice 2 hours after each s.c. injection of [4Y] or PBS. Clotted blood was centrifuged at 13,000xg, serum removed and frozen at -20°C until analysis. Cytokine concentrations were measured using Murine Th1/Th2 10plex FlowCytomix^TM^ Multiplex (eBioscience) according to the manufacturers instructions. Data was acquired on an LSRII (BD) flow cytometer and analyzed using Flow Cytomix Pro 2.4 software (eBioscience).

### Cell isolation

Spleens were disaggregated and red blood cells removed by osmotic lysis. Where indicated, CD4^+^ T cells were isolated using negative magnetic separation with CD4⁺ T cell Isolation Kit II (Miltenyi Biotech) or MagniSort™ Mouse CD4^+^ T cell Enrichment Kit (eBioscience).

### Flow cytometry

Splenocytes were stained with Fixable Viability Dye eFluor® 780 (eBioscience) prior to surface immunostaining. Intranuclear staining (for FoxP3 or cMaf) was performed using FoxP3 Staining Buffers (eBioscience). Intracellular cytokine staining was performed following a 3 hour *in vitro* stimulation in complete RPMI containing 5ng/ml phorbol 12-myristate 13-acetate (PMA) and 500ng/ml ionomycin (both Sigma-Aldrich) in the presence of GolgiStop (BD Biosciences). Cytokine staining was performed using Intracellular Fixation Buffer and Permeabilization Buffer (eBioscience). Data was acquired on an LSR-II or Fortessa X-20 cytometer (BD) and analysed using FlowJo (Treestar).

### RT-PCR

3-5x10^6^ isolated CD4^+^ T cells were stimulated for 18 hours with plate-bound anti-CD3 and anti-CD28 prior to mRNA isolation using an RNeasy Mini Kit, including DNase treatment (QIAGEN). RNA quality and quantity was assessed using a Nanodrop^TM^ 2000 (Thermo Fisher Scientific). Reverse transcription and amplification was carried out using Super-Script III First-strand Synthesis SuperMix for qRT-PCR (Invitrogen). Real-time PCR was performed with QuantiTect SYBR green RT-PCR kits (QIAGEN) using pre-designed Quanti-Tect Primers (Maf, QT01063846; NFIL3, QT00265104; Il10, QT00106169; B2m, QT01149547), using an MJ Opticon Th2 Thermo Cycler (Bio-Rad). The 2^-ΔΔCT^ method was applied to obtain the target gene expression.

### In vitro suppression assay

Splenocytes from Tg4^WT^ and Tg4^KO^ [4Y] and PBS treated mice were cultured in complete RPMI with 10μg/ml [4K] and 20U/ml rhIL-2 (R&D Systems) at a starting concentration of 1x10^6^ cell/ml. After five days, CD4^+^ T cells were isolated by magnetic enrichment. Responder cells were magnetically isolated from naïve Tg4^WT^ mice and labeled with 1mM CellTrace Violet (Life Technologies). 5x10^5^ labeled responder CD4^+^ T cells, 5x10^5^ suppressor CD4^+^ T cells and 1x10^6^ irradiated, sex-matched B10.PL splenocytes (as a source of antigen-presenting cells) were combined with the indicated concentration of [4K] peptide. Cells were cultured for 72 hours before analysis by flow cytometry. Division indices were computed using FlowJo.

### In vivo suppression assay

CD4^+^ T cells isolated from splenocytes of naïve Tg4^WT^ mice were labeled with 10μM Cell Proliferation Dye eFluor® 450 (eBioscience). 1x10^7^ labeled cells were transferred in a volume of 200μl to PBS or [4Y] treated Tg4^WT^ or Tg4^KO^ mice by intraperitoneal injection. After 48 hours, mice were challenged with 80μg [4Y] s.c. and a further 48 hours later spenocytes were analyzed by flow cytometry.

### Statistical analysis

Graphs were constructed and data tested for statistical significance in GraphPad Prism version 6.0.

## Results

### Tg4^KO^ mice have lower serum IL-10 levels following s.c. [4Y] treatment

**Fig 1:**
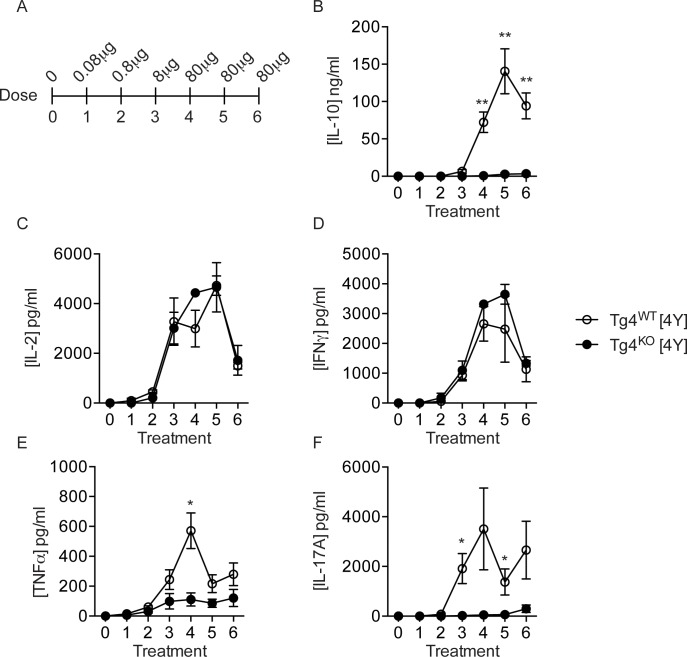
Serum cytokine concentrations in Tg4^WT^ and Tg4^KO^ mice over the course of [4Y] treatment. **(A)** Experimental design. Escalating doses of MBPAc1-9[4Y] peptide were administered subcutaneously to mice every 3–4 days. (**B-F**) Concentrations of IL-10, IL-2, IFNγ, TNFα and IL-17A in serum from peripheral blood taken two hours after each [4Y] treatment in Tg4^WT^ (open circles) and Tg4^KO^ (closed circles) mice. Plots show the average of four animals +/- standard error of the mean (SEM) representing one experiment of three performed. *p<0.01 **p<0.001 by Student’s two-tailed T test.

We administered a previously-described regimen of escalating s.c. doses of [4Y] peptide to Tg4^WT^ and Tg4^KO^ mice (Fig 1A) and measured the concentration of selected cytokines in peripheral blood serum two hours after each treatment, the point of peak cytokine production following peptide administration [1,15]. Most strikingly, concentrations of IL-10 were significantly (up to 100-fold) lower in Tg4^KO^ mice than Tg4^WT^ (Fig 1B). The serum concentration of both IFNγ and IL-2 was equivalent in Tg4^WT^ and Tg4^KO^ mice, both showing a peak at the early 80μg doses which was reduced by the final dose, as shown previously [1] (Fig 1C and 1D). In contrast, serum IL-17A and TNFα concentrations were significantly lower in Tg4^KO^ mice (Fig 1E and 1F). We observed no significant difference in the number of CD4^+^ T cells or the proportion of non-viable cells recovered from spleens of [4Y] treated Tg4^WT^ or Tg4^KO^ mice (S1 Fig), arguing against differences in T cell expansion or cell death between Tg4^WT^ and Tg4^KO^ mice as the cause of reduced serum IL-10 concentrations in Tg4^KO^ mice.

### Lower proportion of IL-10^+^ CD4^+^ T cells and reduced expression of tolerance-associated markers in [4Y] treated Tg4^KO^ mice

**Fig 2:**
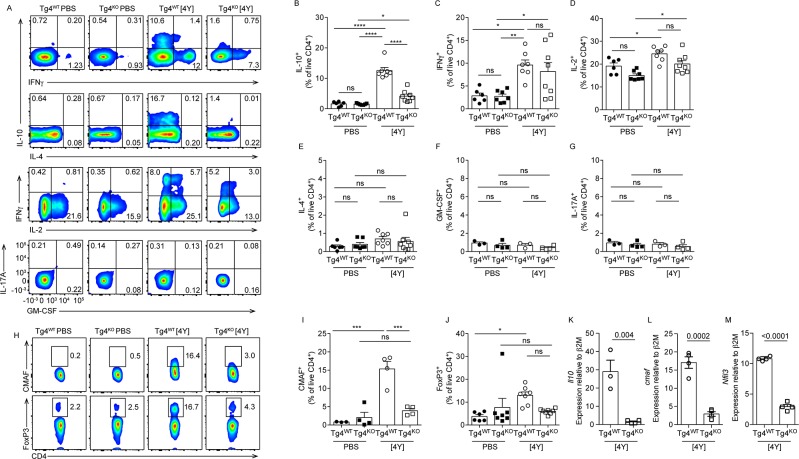
Tg4^KO^ mice show reduced induction of IL-10^+^ T cells and tolerance-associated transcription factors following [4Y] treatment. (**A**) Representative flow cytometry plots and plots of all data (**B-G**) showing production of selected cytokines by splenic CD4^+^ T cells from Tg4^WT^ and Tg4^KO^ mice treated with [4Y] or PBS following *ex vivo* restimulation with PMA and ionomycin. (**H**) Representative flow cytometry plots and plots of combined data (**I-J**) of FoxP3 and cMaf expression in splenic CD4^+^ T cells from Tg4^WT^ and Tg4^KO^ mice treated with [4Y] or PBS. All flow cytometry data is gated on live (viability dye^-^) CD4^+^ cells. (K-M) Expression of *Il10*, *cmaf* and *Nfil3* in magnetically-isolated CD4^+^ splenocytes from Tg4^WT^ and Tg4^KO^ treated with [4Y], restimulated for 18 hours *ex vivo* with anti-CD3ε and CD28, as measured by RT-PCR. Plots F, G, K-M show data from one experiment with 3–4 mice per group. All other plots show the data from two experiments combined, with a total of 6–8 mice per group. All plots show the mean +/- SEM with each point representing data from one animal. *p<0.05, **p<0.01, ***p<0.001, ****p<0.0001, ns p>0.05 assessed by ANOVA with Tukey’s correction for multiple comparisons (plots B-G and I-J). p values shown in K-M were calculated by two-tailed Student’s T test.

We next sought to further characterize the T cell compartment in Tg4^WT^ and Tg4^KO^ mice treated with [4Y] or PBS. Intracellular cytokine staining showed no difference in the proportion of splenic CD4^+^ T cells expressing IL-10, IFNγ, IL-2, IL-4, GM-CSF or IL-17A in Tg4^WT^ and Tg4^KO^ following PBS treatment. There was also no significant difference in the proportion of IFNγ^+^, IL-2^+^, IL-4^+^, GM-CSF^+^ or IL-17A^+^ CD4^+^ T cells in the spleen of either [4Y] treated Tg4^WT^ or Tg4^KO^ mice (Fig 2C–2G). In contrast, the proportion of IL-10^+^ CD4^+^ T cells among Tg4^KO^ splenocytes was significantly lower than in Tg4^WT^ (4.1% +/- 0.7 verses 12.6% +/- 1.0), as was the mRNA expression of *il10* in isolated Tg4^KO^ CD4^+^ T cells (Fig 2B and 2K).

We and others have previously identified transcription factors and cell-surface proteins associated with tolerance and specifically with IL-10^+^ CD4^+^ T cells [1,16]. Following [4Y] treatment, there was a significantly lower proportion of CD4^+^ T cells expressing the tolerance-associated transcription factors cMaf and FoxP3 in Tg4^KO^ splenocytes relative to Tg4^WT^ (Fig 2H–2J). Expression of *cmaf* and *nfil3* (*e4bp4*) mRNA was also approximately 10-fold lower in isolated CD4^+^ T cells from [4Y] treated Tg4^KO^ relative to Tg4^WT^ (Fig 2L and 2M).

**Fig 3:**
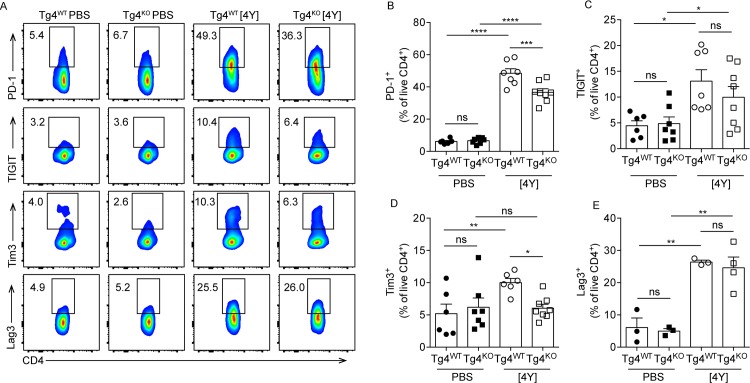
Tg4^KO^ mice express lower levels of tolerance-associated surface markers following [4Y] treatment. (**A**) Representative flow cytometry plots and plots of combined data (**B-E**) showing production of selected cell surface markers by splenic CD4^+^ T cells from Tg4^WT^ and Tg4^KO^ mice treated with [4Y] or PBS. All flow cytometry data is gated on live (viability dye^-^) CD4^+^ cells. Shown is the data from two experiments combined, with a total of 6–8 mice per group. All plots show the mean +/- SEM with each point representing data from one animal. *p<0.05, **p<0.01, ***p<0.001, ****p<0.0001, ns p>0.05 assessed by ANOVA with Tukey’s correction for multiple comparisons.

As previously described [1], the co-inhibitory molecules PD-1, TIGIT, Tim3 and Lag3 were all induced on Tg4^WT^ CD4^+^ T cells following treatment with [4Y] (Fig 3). PKCθ-deficiency affected the expression of these proteins in different ways. Lag3, TIGIT and PD-1 were all induced on Tg4^KO^ mice following [4Y] treatment (Fig 2), although the proportion of CD4^+^ T cells expressing PD-1 was lower in [4Y] treated Tg4^KO^ mice than Tg4^WT^ (Fig 3B). In contrast, Tim3 was not induced in Tg4^KO^ mice following [4Y] treatment (Fig 3D).

### CD4^+^ T cells from [4Y] treated Tg4^KO^ mice do not suppress naïve T cell activation

**Fig 4:**
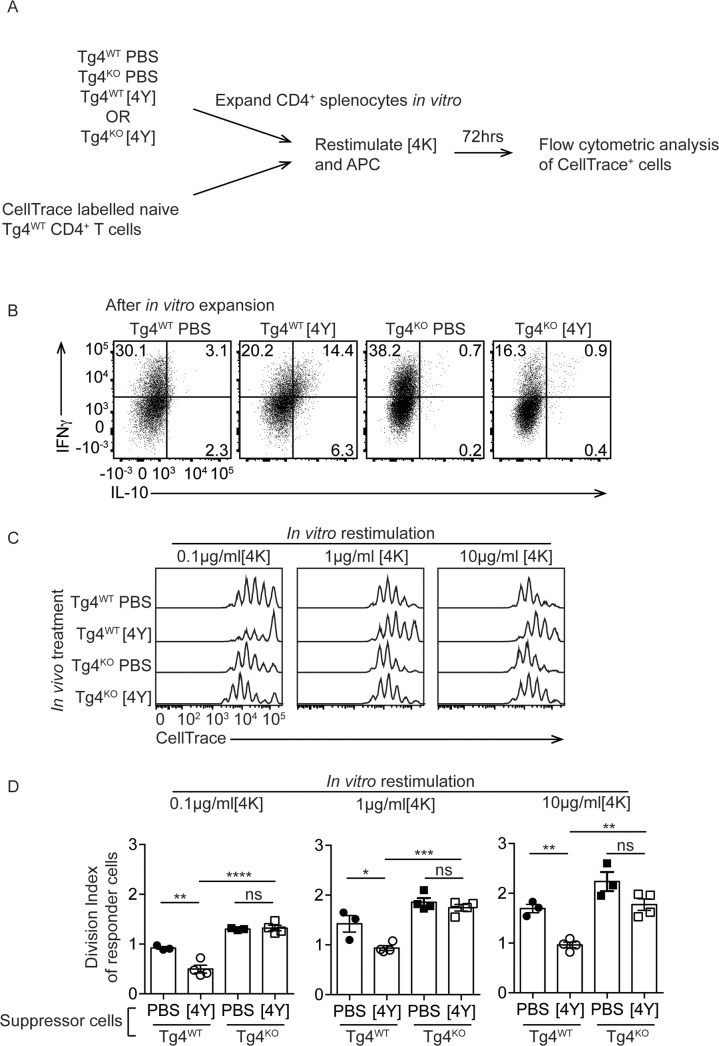
CD4^+^ T cells from [4Y] treated Tg4^KO^ mice do not suppress the proliferation of naïve Tg4^WT^ T cell *in vitro*. (**A**) Experimental design. Splenocytes from Tg4^WT^ and Tg4^KO^ mice, treated with [4Y] or PBS, were expanded *in vitro* with MBPAc1-9[4K] and rhIL-2 for seven days before co-culture with CellTrace-labeled CD4^+^ T cells isolated from naïve Tg4^WT^ mice. (**B**) Examples of the secretion of IL-10 and IFNγ by expanded CD4^+^ cells from the indicated mice, following restimulation with PMA and ionomycin. (**C, D**) Representative flow cytometry data and the computed Division Indices of naïve Tg4^WT^ cells when stimulated with 0.1, 1 or 10μg/ml [4K] and co-cultured with CD4^+^ splenocytes from Tg4^WT^ and Tg4^KO^ mice which had been treated with [4Y] or PBS. Gated on live, CD4^+^, CellTrace^+^ cells. All plots show the mean value +/- SEM. Each point represents data from one [4Y] or PBS-treated mouse assayed individually *in vitro* and is representative of two experiments. *p<0.05, **p<0.01, ns p>0.05 assessed by ANOVA with Tukey’s correction for multiple comparisons.

A characteristic of IL-10^+^ T cells induced by treatment with [4Y] is the ability to suppress the priming of naïve CD4^+^ T cells [1,15] and we sought to test the suppressive capacity of Tg4^KO^ CD4^+^ cells from [4Y] treated mice. CD4^+^ T cells from Tg4^WT^ and Tg4^KO^ mice treated with PBS or [4Y] were first expanded by culturing them for five days with 10μg/ml [4K] and 20U/ml rhIL-2 (as illustrated in Fig 4A). The addition of exogenous IL-2 reverses the anergy of the tolerant T cells, promoting IL-10 secretion, and previous studies have shown that restimulation is necessary to observe optimal suppressive effects *in vitro* [15]. The proliferation of [4Y] treated Tg4^WT^ and Tg4^KO^ cells following restimulation in the presence of IL-2 was equivalent (S2 Fig). The proportion of IL-10^+^ CD4^+^ T cells after expansion is shown in Fig 4B and in each condition is comparable to the proportion see directly *ex vivo* (Fig 2B).

The ability of each T cell population to suppress naive T cells responses was assessed by co-culturing CellTrace Violet labeled naïve Tg4^WT^ CD4^+^ T cells, expanded CD4^+^ T cells from treated mice and irradiated B10.PL (wildtype) splenocytes as a source of antigen presenting cells at a ratio of 1:1:2 with 0.1, 1 or 10μg/ml [4K] peptide. After 72 hours, the proliferation of the Tg4^WT^ responders was assessed by flow cytometry (as illustrated in Fig 4A). Tg4^WT^ [4Y] treated cells significantly suppressed the proliferation of the responder cells relative to Tg4^WT^ PBS treated cells at all concentrations of antigen tested (Fig 4C and 4D). In contrast, [4Y] treated Tg4^KO^ cells failed to suppress responder cell proliferation, regardless of the concentration of antigen. We considered that Tg4^KO^ cells could fail to suppress if they had died upon restimulation. Analysis of the proportion of live cells recovered at the end of the co-culture demonstrated that the [4Y] treated Tg4^WT^ and Tg4^KO^ cells were similarly viable following restimulation (S2B Fig).

**Fig 5:**
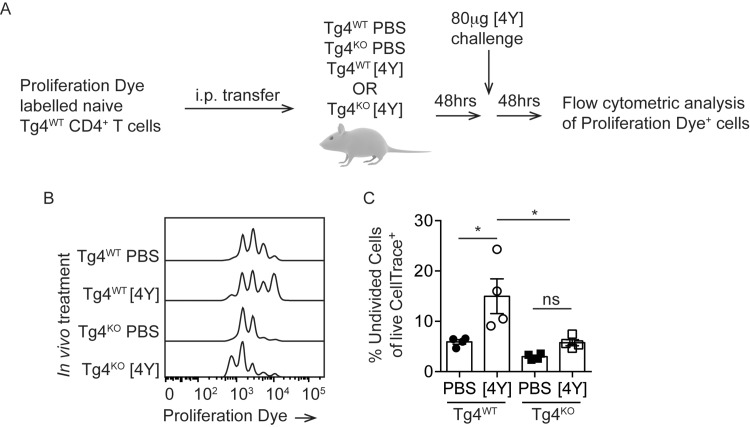
PKCθ is required for induction of a suppressive environment *in vivo*. (**A**) Experimental design. Cell Proliferation Dye-labeled CD4^+^ T cells from naïve Tg4WT mice were adoptively transferred to Tg4^WT^ and Tg4^KO^ mice, pretreated with [4Y] or PBS. After 48 hours, mice were challenged with 80μg of [4Y] and the division of the transferred Tg4^WT^ cells was measured by flow cytometry after a further 48 hours. (**B**) Example flow cytometry data and (**C**) plotted data from all mice showing the proportion of transferred Tg4^WT^ cells which remained undivided following [4Y] challenge under each pre-treatment condition. Shown is the mean +/- SEM. Each data point represents one [4Y] or PBS-treated recipient mouse which were assayed in a single experiment. *p<0.05 assessed by ANOVA with Tukey’s correction for multiple comparisons.

Previous work has demonstrated discrepancies between mechanisms of Treg-mediated suppression when assayed *in vitro* and *in vivo* [15]. We therefore tested the suppressive capacity of [4Y] treated Tg4^KO^ cells *in vivo*. Cell Proliferation Dye-labeled naïve Tg4^WT^ cells were adoptively transferred into Tg4^WT^ and Tg4^KO^ mice pre-treated with [4Y] or PBS. After 48 hours, the mice were challenged with 80μg [4Y] and a further 48 hours later the proliferation of the responder cells recovered from the spleen was assessed by flow cytometry (as illustrated in Fig 5A). A challenge dose of 80μg of 4Y was chosen as we have demonstrated previously that suppression observed at this dose of antigen correlates with multiple other measures of regulatory function, including amelioration of EAE [1]. In PBS-treated Tg4^WT^ mice the proportion of undivided responder cells was 5.9% +/- 0.9 whereas pre-treatment with [4Y] resulted in significant suppression of the responder cells in Tg4^WT^ mice with 15.0% +/-3.5 of cells remaining undivided. Pre-treating Tg4^KO^ mice with [4Y] did not induce suppression of the transferred responder cells and the proportion of undivided cells in the [4Y] treated Tg4^KO^ mice was not significantly higher than in PBS-treated Tg4^KO^ mice and was significantly lower than in [4Y] treated Tg4^WT^ mice (Fig 5B and 5C).

## Discussion

The aim of this study was to determine if the signaling protein PKCθ plays a role in the induction of suppressive IL-10^+^ T cells during a tolerance induction protocol of self-antigen administration. IL-10 plays an essential role in the regulation of immune responses and it is therefore important to understand the mechanisms by which it is induced [17]. Furthermore, if PKCθ is to be considered a suitable drug target in immune-mediated diseases [7,9], it is essential to understand the implications that inhibiting this kinase may have on all immunoregulatory processes.

We demonstrate that PKCθ knock out TCR-transgenic mice fail to upregulate IL-10 following the sequential administration of escalating doses of a high-affinity variant of the relevant cognate antigen, a well-described protocol for the induction of IL-10^+^ T cells [1,2,18]. Tg4^KO^ mice show significantly reduced serum concentrations of IL-10 and IL-17A following peptide treatment, but concentrations of the Th1-associated cytokines IFNγ and IL-2 are equivalent in Tg4^WT^ and Tg4^KO^ mice. We also observe a reduced proportion of IL-10^+^ CD4^+^ splenocytes in Tg4^KO^ mice compared to Tg4^WT^, but proportions of IFNγ^+^ and IL-2^+^ CD4^+^ T cells are unaffected by PKCθ deficiency. This is consistent with observations that PKCθ is required for the differentiation of Th17 but not Th1 cells [5,19]. The transcription factor cMaf is essential for IL-10 transcription in macrophages and upregulation of cMaf is associated with IL-10 expression in CD4^+^ T cells [1,20]. Similarly, FoxP3 and Nfil3 have both been shown to regulate IL-10 transcription [21]. cMaf is strongly induced in [4Y] treated Tg4^WT^ mice, but is not upregulated in Tg4^KO^ mice CD4^+^ T cells. FoxP3 is induced in a small proportion of Tg4^WT^ cells, but not in Tg4^KO^ cells. Similarly, mRNA expression of Nfil3 is approximately 4-fold lower in [4Y] treated Tg4^KO^ cells compared to Tg4^WT^. As *il10* mRNA levels are also significantly reduced in Tg4^KO^ cells, it suggests that the transcription factors required to drive expression of IL-10 are not induced in Tg4^KO^ cells and that this contributes to the reduced levels of IL-10 observed.

The mechanism by which Nfil3 is induced in CD4^+^ T cells remains unknown. In natural killer cells, induction of Nfil3 is dependent on 3′-phosphoinositide–dependent kinase 1 (PDK1) [22], a known substrate of PKCθ [22,23].This suggests a possible pathway downstream of PKCθ that could lead to Nfil3 expression in CD4^+^ T cells. It is similarly unclear what links PKCθ activation to the induction of cMaf expression. Under conditions favoring the development of Th17 cells, induction of cMaf in CD4^+^ cells is dependent upon Stat3 [24] and PKCθ has been shown to induce expression of Stat3 via NFκB and AP-1 [5], providing a possible link between PKCθ and cMaf.

We also observed altered expression of tolerance-associated co-inhibitory molecules in Tg4^KO^ CD4^+^ cells following [4Y] treatment. Although PD-1 was induced on Tg4^KO^ CD4^+^ T cells, it was on a lower proportion of cells than in Tg4^WT^ mice. Tim3 was not induced on Tg4^KO^ cells but expression of Lag3 and TIGIT was, on average, unaffected by PKCθ deficiency. The mechanisms of action and the relative roles of these co-inhibitory receptors is poorly defined [25], but this data suggest that each may be induced via different stimuli, some dependent of PKCθ and some not. The failure of Tg4^KO^ cells to upregulate Tim3 may be explained by the deficient *Nfil3* expression in these cells as Nfil3 has been shown to be required for Tim3 expression in IL-27-induced regulatory cells [26].

We have previously demonstrated that [4Y] treatment protects Tg4^WT^ and B10.PL non-TCR transgenic mice from experimental autoimmune encephalomyelitis (EAE) [1]. Due to the impact of PKCθ deficiency on CD4^+^ T cell priming and Th17 differentiation, PKCθ knockout mice are generally resistant to EAE induction [27]. These confounding factors make it difficult to test the ability of Tg4^KO^ [4Y]-induced IL-10^+^ T cells to suppress T cell activation in this disease model. Instead we chose to assess the capacity of these cells to prevent Tg4^WT^ cell activation in *in vitro* co-cultures and in an adoptive transfer model of suppression. We demonstrate that [4Y] treated Tg4^KO^ T cells are unable to suppress the proliferation of naïve Tg4^WT^ cells in both *in vitro* in co-cultures and *in vivo* following adoptive transfer of responder cells to pre-treated Tg4^KO^ mice. PKCθ is required for efficient FoxP3^+^ Treg induction [28] and functions to modulate the activity of thymically-derived Treg [6]. Given these observations we cannot exclude the possibility that altered induction or function of FoxP3^+^ Treg is responsible for the reduced suppressive activity we observe in T cells from 4Y-treated Tg4^KO^ mice. However, we have previously excluded a role for FoxP3+ Treg in suppression following 4Y treatment [29], supporting our hypothesis that defective IL-10 induction from Tr1-like cells is the cause of reduced suppression in Tg4^KO^ mice.

We observe that CD4^+^ T cells from [4Y] treated Tg4^KO^ mice lack only some of the tolerance-associated characteristics of [4Y] treated Tg4^WT^ cells (of those measured, specifically IL-10, cMaf, Nfil3 and Tim3) and this may provide information about which molecules mediate the cells suppressive ability. Induction of Lag3 and TIGIT is unaffected by PKCθ deficiency, but the cells are unable to suppress proliferation of the responders, suggesting that these proteins do not play a significant role in the suppression of naïve T cell responses in this system.

When interpreting the experiments presented here it is important to consider the role that PKCθ plays in all aspects of immune biology and how this may impact the induction of IL-10^+^ T cells. For example, PKCθ is required for efficient positive selection of T cells in the thymus [30]. Even in the context of a transgenic TCRαβ a second endogenous α-chain can be selected and variation in this repertoire as a result of PKCθ deficiency may alter the behavior of CD4^+^ T cells in the periphery. Until recently, PKCθ expression was believed to be restricted to the lymphoid lineage. A recent study has described expression of PKCθ mRNA in macrophages and defined a role for PKCθ in the response of macrophages to infection [31]. This opens the previously unappreciated possibility that PKCθ-deficiency may impact other aspects of the myeloid lineage, innate-derived cytokine production and potentially antigen presentation.

This work presented here demonstrates that PKCθ is required for the efficient induction of IL-10 in effector T cells following administration of an escalating dose of self-antigen to TCR-transgenic mice. This should be considered when contemplating PKCθ as a suitable drug target for promoting immune tolerance and the impact of PKCθ inhibitors, including sotrastaurin, on the induction of IL-10^+^ CD4 T cells should be investigated.

## Supporting information

S1 Fig Related to Figs 1–3. Tg4^WT^ and Tg4^KO^ mice have a similar number and viability of CD4^+^ T cells following [4Y] treatment. (A) The number of CD4^+^ T cells per spleen in Tg4^WT^ and Tg4^KO^ mice treated with [4Y] or PBS. (B) The proportion of viability dye (Fixable Viability Dye eFluor780) positive cells in spleens from Tg4^WT^ and Tg4^KO^ mice treated with [4Y] or PBS. Both plots show the mean +/- SEM with each point representing data from one animal. **p<0.01, ns p>0.05 assessed by ANOVA with Tukey’s correction for multiple comparisons
(TIF)

S2 Fig Related to Fig 4. **(A)** CD4^+^ T cells from [4Y] treated Tg4^WT^ and Tg4^KO^ expand similarly in response to antigen and IL-2. Splenocytes from Tg4^WT^ and Tg4^KO^ mice treated with [4Y] were stimulated in vitro with 10μg/ml [4K] peptide +/- 20U/ml rhIL-2 as indicated. Proliferation was measured by incorporation of ^3^H thymidine, which was added 72 hours after restimulation. The plot shows the mean values from four mice per group, each assayed in triplicate (a total of 12 data points per group), +/- SEM. (**B**) The proportion of viable (Fixable Viability Dye eFluor780 negative) suppressor cells (Cell Proliferation Dye negative, from Tg4^WT^ or Tg4^KO^ mice treated with PBS or [4Y]) recovered after 72 hours of co-culture with naïve responder cells and the indicated concentration of [4K] peptide. The plots show the mean values from 3–4 mice per group +/- SEM. ****p<0.0001, ns p>0.05 assessed by ANOVA with Tukey’s correction for multiple comparisons.
(TIF)
