# Supplementary figures and images for: Protein kinase C theta is required for efficient induction of IL-10-secreting T cells
Source: PLoS One. 2017 Feb 3;12(2):e0171547. doi: 10.1371/journal.pone.0171547 (PMC5291537; doi:10.1371/journal.pone.0171547)

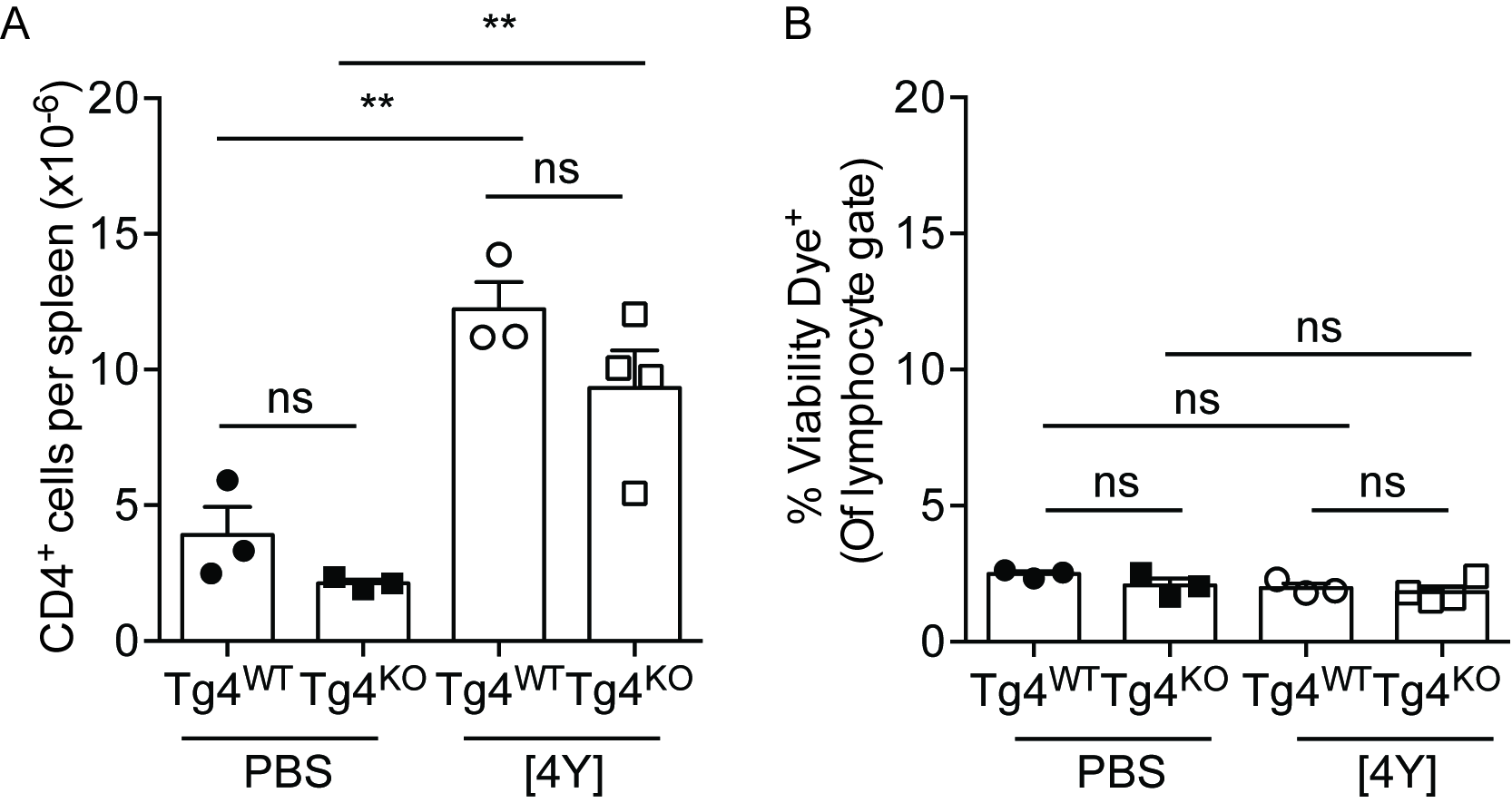

Supplement: S1 Fig — Tg4WT and Tg4KO mice have a similar number and viability of CD4+ T cells following [4Y] treatment. (A) The number of CD4+ T cells per spleen in Tg4WT and Tg4KO mice treated with [4Y] or PBS. (B) The proportion of viability dye (Fixable Viability Dye eFluor780) positive cells in spleens from Tg4WT and Tg4KO mice treated with [4Y] or PBS. Both plots show the mean +/- SEM with each point representing data from one animal. **p<0.01, ns p>0.05 assessed by ANOVA with Tukey’s correction for multiple comparisons (TIF) [file pone.0171547.s001.tif]

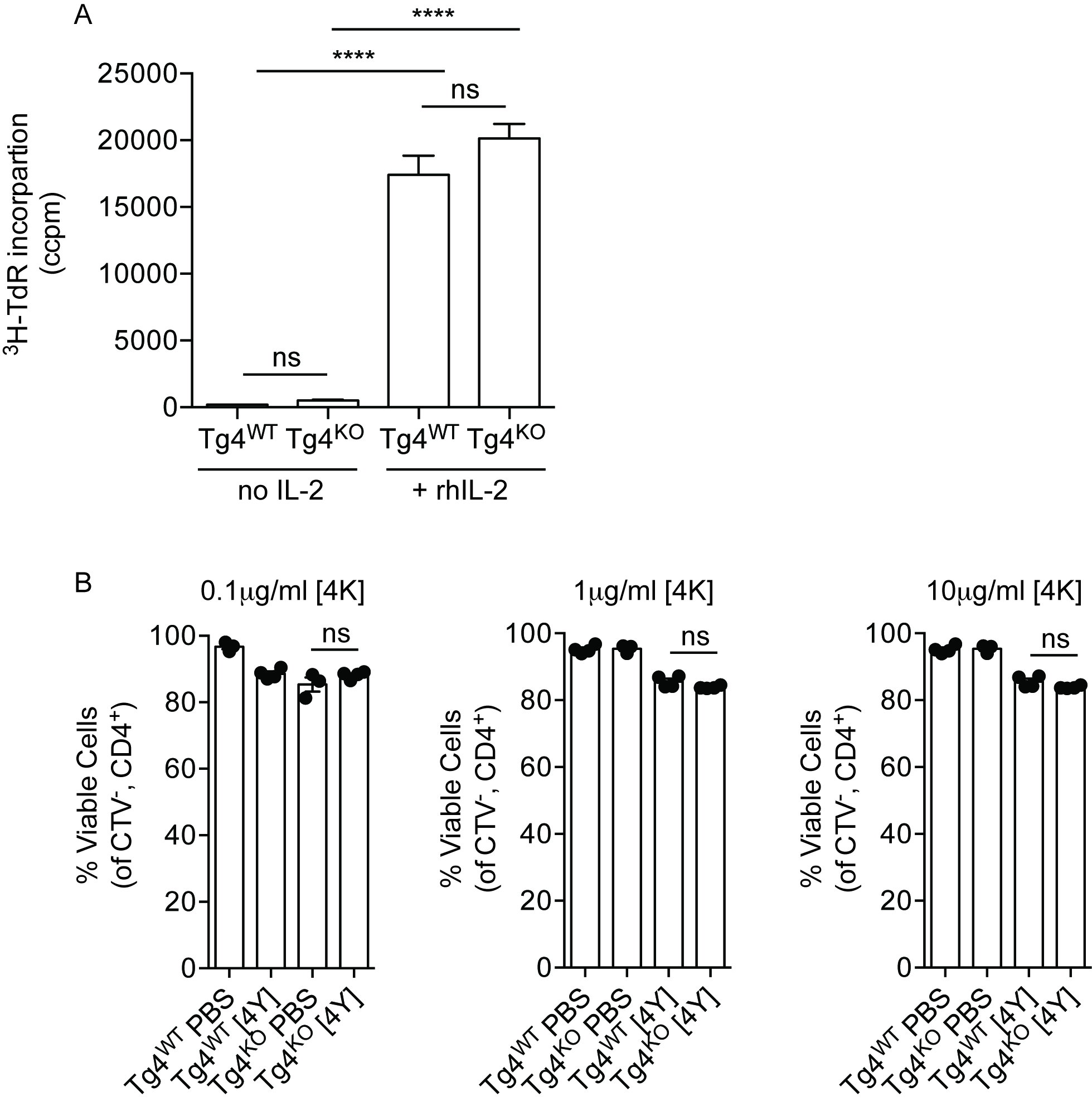

Supplement: S2 Fig — (A) CD4+ T cells from [4Y] treated Tg4WT and Tg4KO expand similarly in response to antigen and IL-2. Splenocytes from Tg4WT and Tg4KO mice treated with [4Y] were stimulated in vitro with 10μg/ml [4K] peptide +/- 20U/ml rhIL-2 as indicated. Proliferation was measured by incorporation of 3H thymidine, which was added 72 hours after restimulation. The plot shows the mean values from four mice per group, each assayed in triplicate (a total of 12 data points per group), +/- SEM. (B) The proportion of viable (Fixable Viability Dye eFluor780 negative) suppressor cells (Cell Proliferation Dye negative, from Tg4WT or Tg4KO mice treated with PBS or [4Y]) recovered after 72 hours of co-culture with naïve responder cells and the indicated concentration of [4K] peptide. The plots show the mean values from 3–4 mice per group +/- SEM. ****p<0.0001, ns p>0.05 assessed by ANOVA with Tukey’s correction for multiple comparisons. (TIF) [file pone.0171547.s002.tif]
